# Mapping play-based interventions for children with disabilities in LMICs: a scoping review on cultural relevance, implementation, and impact

**DOI:** 10.1016/j.eclinm.2025.103444

**Published:** 2025-09-02

**Authors:** Veronika Reichenberger, Alberta S.J. van der Watt, Quinette Louw, Soraya Seedat, Tracey Smythe

**Affiliations:** a International Centre for Evidence in Disability, Department of Population Health, PHP, LSHTM, UK; b Department of Psychiatry, Faculty of Medicine & Health Sciences, Stellenbosch University, Tygerberg, South Africa; c Department of Health and Rehabilitation Sciences, Faculty of Medicine & Health Sciences, Stellenbosch University, Tygerberg, South Africa; d SAMRC Genomics and Brain Disorders Research Unit, Department of Psychiatry, Stellenbosch University, Tygerberg, South Africa

**Keywords:** Children, Caregivers, Developmental disability, Intervention, Social inclusion, Scoping review

## Abstract

**Background:**

Over 90% of children with disabilities live in low- and middle-income countries (LMICs) and experience worse health outcomes than their peers. Play-based interventions may improve the well-being and quality of life of children with disabilities and their caregivers. We aimed to identify and map the evidence on play-based interventions for children with disabilities in LMICs.

**Methods:**

Following a systematic search of Medline, Embase, PsycINFO, African Journals Online, and LILACS, we screened 2577 studies for inclusion in October 2024. We used thematic analysis to summarise the data.

**Findings:**

Twenty eligible studies were identified that targeted children with disabilities, aged 2–12 years. Most studies (55%) focused on how play supports therapy, communication, and skills development, while fewer studies (15%) explored feasibility, developmental outcomes, or parental support. A substantial proportion of studies took place in upper-middle-income countries (60%) and 50% were from Central and South America. Many interventions were delivered by researchers (55%), with some involving parents (25%), community members (5%), or health professionals (10%). Cultural and contextual adaptations were noted in 40% of studies, and these changes were typically confined to surface features such as use of local languages, traditional games, and culturally relevant illustrations. While most studies reported positive outcomes, concerns were raised about limited parental engagement and challenges in adaptation. Only one study conducted a cost analysis. Ten grey literature resources met inclusion criteria. These resources offered practical guidance, mainly for caregivers and educators, on making play accessible for children with disabilities. Most emphasised play's role in development and inclusion, with 80% providing specific strategies, though none addressed cultural adaptation.

**Interpretation:**

Play-based interventions have the potential to enhance therapy, communication, and social inclusion, although gaps remain in understanding their feasibility and scalability. The lack of economic evaluations also limits the understanding of how these interventions can be implemented in low-resource settings. Our review highlights critical gaps in play-based interventions for children with disabilities in LMICs. Despite growing interest since the UNCRPD, play remains under-researched, with most studies being small-scale, short-term, and lacking cost analysis. Interventions rarely involved communities or addressed cultural relevance in depth. Future research should prioritise inclusive, community-driven, and culturally adapted approaches. Clearer documentation, economic evaluations, and rigorous methods are needed to ensure effective, scalable, and meaningful support. Promoting culturally grounded play can improve social inclusion, development, and dignity for children with disabilities.

**Funding:**

The scoping review was supported by the United Kingdom 10.13039/501100000265Medical Research Council (UKRI165) awarded to Prof Tracey Smythe in collaboration with the London School of Hygiene & Tropical Medicine.

**Research in context**

**Evidence before this study**

Carrington et al. (2023) conducted a systematic review of play interventions for children with disabilities that identified few studies in LMICs, with three studies from India and one from Ethiopia. This review found that data inconsistency and diverse study designs reduced the overall certainty and applicability of play as a therapeutic intervention. We searched Embase, Medline and PsycINFO on Oct 1, 2024 for trials involving play among children with disabilities in LMICs using the following combination of keywords: “disability” AND “play” AND “low and middle income”. We found no trials of play interventions delivered in the community for children with disabilities.

**Added value of this study**

To our knowledge, this is the first review of play-interventions for children with disabilities in LMICs. We have provided a comprehensive summary of published evidence. We identified that most studies focused on children with multiple impairments. Play interventions were primarily delivered by researchers, with minimal involvement of parents or community members. Fewer than half of the studies reported considering culture or context, and none conducted a comprehensive cost analysis, which limits insights into financial feasibility and scalability in LMICs.

**Implications of all the available evidence**

The limited evidence on play interventions for children with disabilities in LMICs reflects broader structural inequities in intervention development. There is a dominance of researcher-led play initiatives, rather than community-driven approaches, which may impact sustainability, local ownership, and cultural relevance. There is urgent need for cost analyses and participatory methodologies to evaluate feasibility and scalability of play interventions within diverse socio-economic contexts.

## Introduction

Worldwide, there are nearly 240 million children with disabilities, 90% of whom live in low- and middle-income countries (LMICs).^1^ The Convention on the Rights of Persons with Disabilities defines living with a disability as having a long-term physical, mental, intellectual or sensory impairment that, in interaction with the environment hinders one's participation in society on an equal basis with others.^2^ Children with disabilities experience worse health and are at greater risk of mortality than their peers.^3^ Yet, most children with disabilities in LMICs do not receive formal support^4^ and efforts to expand support are hindered by the scarcity of skilled human resources, fragmented healthcare systems,^5^ and already overburdened caregivers.^6^ These caregivers often experience anxiety, depression, physical exhaustion, stigma, negative attitudes from others, social exclusion, and discrimination.^6^, ^7^, ^8^, ^9^

Whilst play is essential for the development and well-being of all children, children with disabilities are often excluded from play opportunities^10^ and a recent systematic review identified few interventions to address their social inclusion or well-being.^11^ Play enables children, including those with disabilities, to explore emotions, develop communication skills, and build confidence and healthy coping mechanisms essential for thriving in adolescence and beyond.^12^^,^^13^ Recognising this gap, the WHO and UNICEF recommend supporting children with disabilities through inclusive policies, targeted programmes, capacity development, public awareness, and data collection.^14^ However, barriers to accessing and delivering interventions vary locally and “top-down” approaches may not be sufficient for enhanced inclusive play opportunities. Contextual factors influence the lived experiences of children with disabilities and their caregivers,^15^ and must be considered when developing play-based interventions to avoid misunderstandings, resistance, or harm.^16^^,^^17^ Further, culturally sensitive play interventions can leverage existing community resources, such as local caregivers, educators, and social support systems or networks, to build capacity and long-term sustainability.^18^ These play-based interventions may actively engage children with disabilities in meaningful play experiences, and foster their development while addressing local barriers through community-driven, participatory approaches.

Lived experiences are also shaped by culture.^15^ Notably, play is shaped by unique traditions, beliefs, and practices within each community, influencing children's early experiences and social interactions.^19^^,^^20^ Accordingly, cultural beliefs, gender norms, religious beliefs, and community and societal attitudes toward disability can impact children's inclusion in play activities.^21^ For example, disability may be viewed as a source of shame or stigma, leading families and communities to isolate people with disabilities or limit children's interactions with their peers.^22^^,^^23^ Further, caregivers' perceptions and concerns about safety or potential harm can restrict play, particularly if the local understanding of disability limits the types of activities considered safe or appropriate.^24^, ^25^, ^26^ Thus, play programmes need to reflect local values and perspectives on disability to ensure that the interventions better resonate with caregivers and communities, fostering acceptance and meaningful engagement,^13^ reducing stigma, and promoting social inclusion of children with disabilities^21^ and their caregivers. For example, culturally relevant play-based interventions that incorporate traditional games, local stories, and familiar materials have been found to increase children's connection with the intervention^27^ and enhance engagement, learning, and social and emotional development.^28^ Culturally adapted play interventions are also more likely to be accepted and supported by communities, teachers, and caregivers, creating an enabling environment for children's development^29^; thereby enhancing the interventions' appropriateness, effectiveness, and sustainability across diverse populations and geographic settings.^15^

Play is a fundamental right for all children, recognised in both the UN Convention on the Rights of the Child (Article 31) and the UN Convention on the Rights of Persons with Disabilities. Yet, most evidence on play-based interventions is rooted in high-income settings, often overlooking the social, cultural, and material realities of children in LMICs. There is a growing recognition that child development must be understood within local contexts, where play takes on different meanings, forms, and functions. However, a recent systematic review of the effectiveness of play-based interventions for children with disabilities only identified eligible studies from India, Ethiopia, and China.^13^

Despite the recognised importance of play for child development, there is a dearth of literature regarding culturally appropriate play-based interventions for children with disabilities in LMICs. We aimed to map and evaluate existing evidence to inform culturally appropriate play-based programme development for children with disabilities in LMICs. For this review, the term ‘culturally appropriate’ referred to interventions that considered and incorporated the social, linguistic, and cultural norms of the communities they aimed to serve.^17^

## Methods

This scoping review is registered on OSF (https://osf.io/7tr63) and followed the PRISMA Extension for Scoping Reviews (PRISMA-ScR) guideline^30^ and Arksey and O'Malley's framework for scoping reviews.^31^ We conducted the review across five stages: (i) identifying the research question; (ii) identifying relevant studies; (iii) selecting studies; (iv) charting the data; and (v) collating and summarising the results.

### Literature search

Search terms were developed with librarians from the London School of Hygiene & Tropical Medicine and Stellenbosch University and included terms around disability, children, play, and low- and middle-income countries. Medline, Embase, PsycINFO, African Journals Online, and LILACS were systematically searched in October 2024 to identify eligible studies. Additionally, we searched for grey literature using OpenGrey and Google Scholar by reviewing the first 50 citations. Lastly, reference lists of included articles and systematic reviews were screened to identify additional articles for inclusion.

### Inclusion criteria

Full-text articles and grey literature published between 1 September 2004 and 30 October 2024 of any study design (quantitative, qualitative, and mixed methods) were included. Descriptive literature (i.e., without an applied research method) was included if sufficient detail was provided on the intervention, such as participant characteristics, target population, how the interventions were conducted and by whom, and the setting in which the interventions were conducted. Studies in English, French, Spanish, and Portuguese were included.

The grey literature included reports, policy documents, and NGO websites. Documents needed to provide sufficient methodological detail or programmatic insights that were relevant to play-based interventions for children with disabilities in LMICs. Thus, if a website was hosted by an international NGO or in a high-income country with practical applications for low-resourced settings, it was eligible for inclusion.

### Exclusion criteria

Full-text studies undertaken only in high-income countries were excluded. Reviews, opinion pieces, editorials, and non-research-based commentaries in the grey literature were not eligible.

#### Population

The target population was children aged 2–12 years (and/or their caregivers) living in LMICs with a diagnosed, proxy-reported, or self-reported disability of any severity—defined as a broad range of impairments that may affect a child's physical, cognitive, sensory, or social functioning.^32^ During the selected age range, play is developmentally relevant and distinct from structured activities like sport or digital engagement that are more common in adolescent age groups. Studies where only part of the sample met this age range were excluded if age-specific results could not be separated. Studies conducted in LMICs and high-income countries were included if the country-specific results could be disaggregated. We followed the World Bank's 2025 definition of LMICs.^33^

#### Intervention

Any play-based intervention, regardless of cultural or contextual adaptation, was included. For this paper, play was defined as a voluntary, intrinsically motivated activity that involves active engagement, imagination, and exploration, providing opportunities for learning and social interaction.^34^^,^^35^ The interventions could promote any outcome (albeit physical, social, or emotional) and could be delivered by any person in any setting. Studies with or without assessment of any outcome were included. Children (2–12 years old) had to be the target of the intervention. Interventions that only targeted parents, caregivers, or other adults (e.g., teachers) were excluded.

### Screening process

**Fig. 1:**
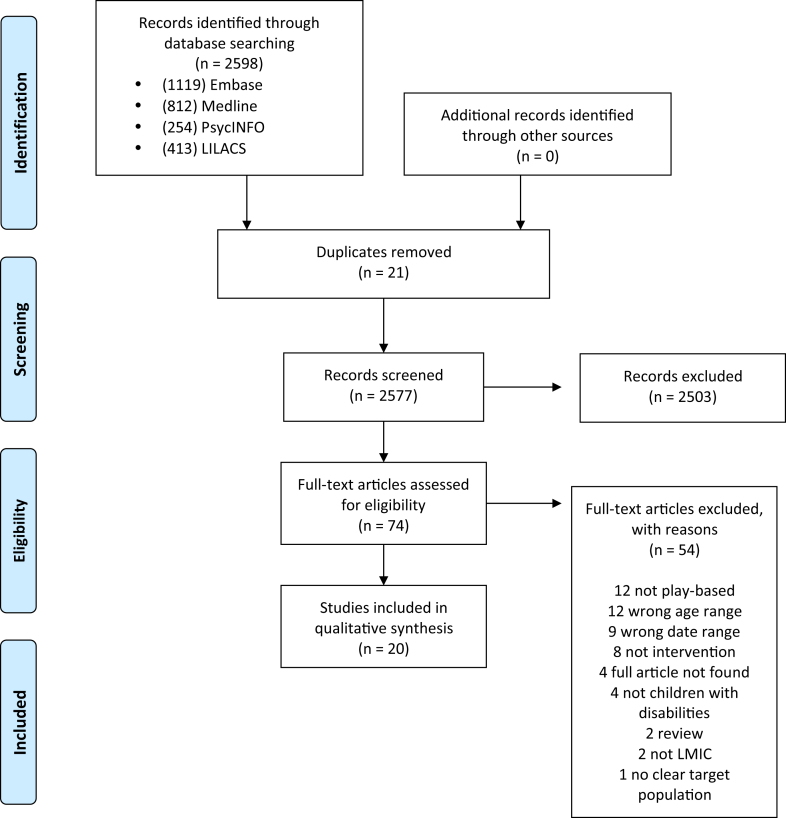
PRISMA flow diagram.

We used Rayyan^36^ to manage the study selection. Two researchers (VR and TS) independently screened all titles and abstracts against the eligibility criteria. Two independent researchers (VR and AvdW) screened eligible full-text articles. Discrepancies at any stage were discussed between the reviewers, and a third reviewer (TS or AvdW) was consulted where necessary. The screening process is depicted in the PRISMA diagram (Fig. 1).

### Data extraction and synthesis

Data were extracted independently by VR and AvDW for each study using a customised form developed in Excel that was piloted on three included studies. VR and AvdW reviewed each other's data extracted. Discrepancies were discussed. The data extracted included:

(i) Study details: Author, year of publication, title of publication, country, aims/objectives, study design).
(ii) Sample and intervention characteristics of the play intervention: Disability type, intended outcome, delivery agent, intervention components, cultural appropriateness.
(iii) Outcomes: Effectiveness, feasibility, acceptability, cost analysis, measurement tools, findings.

Findings from qualitative and mixed-methods studies were synthesised narratively and integrated alongside quantitative results to highlight key themes such as feasibility, cultural adaptation, and user engagement. Given the descriptive nature and limited depth of qualitative data across studies, a separate thematic synthesis was not conducted. Specifically, full-text articles were included in the synthesis, and grey literature was summarised separately.

### Role of the funding source

The funders did not have any role in the development, design, and undertaking of this scoping review. All authors had access to the data and were responsible for the decision to submit this scoping review for publication.

## Results

### Eligible studies

Twenty articles met the inclusion criteria (see Table 1 for a summary of the study and population characteristics). The main reasons for exclusion during the full text screening included the children's age (*n* = 12; 15%), the intervention not being play-based (*n* = 12; 15%), publication date (*n* = 9; 11%), and no data provided on the intervention or no intervention being present (*n* = 8; 10%). Most articles were published in English (*n* = 13, 65%),^37^, ^38^, ^39^, ^40^, ^41^, ^42^, ^43^, ^44^, ^45^, ^46^, ^47^, ^48^, ^49^ with four published in Spanish (20%),^50^, ^51^, ^52^, ^53^ and three published in Portuguese (15%).^54^, ^55^, ^56^

**Table 1:** Summary of article characteristics.

Variable and category	N (%)
Region	
Africa	4 (20%)
Asia	5 (25%)
Central and South America	10 (50%)
Middle East	1 (5%)
Country	
Brazil	4 (20%)
Colombia	3 (15%)
Cuba	1 (5%)
Ethiopia	1 (5%)
Guatemala	1 (5%)
India	2 (10%)
Indonesia	1 (5%)
Iran	1 (5%)
Kenya	1 (5%)
Malawi	1 (5%)
Mexico	1 (5%)
Nigeria	1 (5%)
Pakistan	1 (5%)
Thailand	1 (5%)
Country income status	
Low income	2 (10%)
Lower-middle income	6 (30%)
Upper-middle income	12 (60%)
Child impairment type	
Multiple	15 (75%)
Visual	3 (15%)
Physical	1 (5%)
Not specified	1 (5%)
Outcome measured	
Play and social skills	9 (45%)
Parental wellbeing	2 (10%)
Feasibility and acceptability of play interventions	3 (15%)
Child development	4 (20%)
Disability inclusive play	1 (5%)
Study design^a^	
RCT	3 (15%)
Controlled before-after	3 (15%)
One group before-after, no control	6 (30%)
Longitudinal mixed	2 (10%)
Programme evaluation	3 (15%)
Qualitative—IDI, FGD and/or observation	8 (40%)
Sample size	
<10	10 (50%)
11–20	3 (15%)
21–50	5 (25%)
51–100	2 (10%)

RCT, randomized control trial; IDI, in-depth interview; FGD, focus group discussion.

a Some studies had multiple study designs.

### Study objectives

One study (5%)^37^ examined parental anxieties and how to support parents of children with neurodisabilities or intellectual disabilities. This study also reported on how supporting parents may impact children's development. Eleven studies (55%)^39^^,^^41^^,^^43^^,^^45^, ^46^, ^47^^,^^50^, ^51^, ^52^^,^^54^^,^^56^ were about play and how it helps with therapy, communication, interaction, and skills, such as using adapted toys, group play, and playful strategies for children with cerebral palsy or neurodevelopmental delays. Three studies (15%)^38^^,^^42^^,^^48^ focused on the feasibility and acceptability of play interventions that were adapted to different settings (Kenya, Malawi, and Pakistan), while another three studies (15%)^40^^,^^44^^,^^55^ focused on specific developmental outcomes such as motor skills, vision, and cognitive abilities. One study (5%)^53^ centred on helping visually impaired children with mobility, orientation, and making play more inclusive. One study (5%) did not specify its objective.^49^

### Study characteristics

Ten studies were from Central and South America (50%),^39^^,^^45^^,^^46^^,^^50^, ^51^, ^52^, ^53^, ^54^, ^55^, ^56^ five studies were conducted in Asia (25%),^37^^,^^38^^,^^40^^,^^44^^,^^47^ four in Africa (20%)^41^^,^^42^^,^^48^^,^^49^ and one in the Middle East (5%).^43^ Of these, 12 were in upper-middle income countries (60%),^39^^,^^43^, ^44^, ^45^, ^46^^,^^50^, ^51^, ^52^, ^53^, ^54^, ^55^, ^56^ six were conducted in low-middle income countries (30%),^37^^,^^38^^,^^40^^,^^41^^,^^47^^,^^48^ and two in low-income countries (10%).^42^^,^^49^

Seven studies (35%) used a one-group before-after design without a control group,^37^^,^^40^^,^^45^, ^46^, ^47^, ^48^^,^^55^ and three (15%) were randomised controlled trials.^38^^,^^44^^,^^50^ Others included controlled before-after designs (*n* = 3; 15%),^41^^,^^43^^,^^54^ programme evaluations (*n* = 4; 20%),^42^^,^^51^^,^^53^^,^^56^ and qualitative methodologies (*n* = 2; 10%),^39^^,^^49^ with one study combining methods.^52^ Follow-up periods varied widely: 9 studies (45%) reported no follow-up,^38^, ^39^, ^40^, ^41^^,^^43^^,^^45^^,^^47^, ^48^, ^49^ while others ranged from 2 weeks to 30 months, which reflects variability in study duration and intensity.

The study settings were varied. Eight studies were conducted in schools (40%),^37^^,^^40^^,^^41^^,^^43^^,^^47^^,^^52^, ^53^, ^54^ four in community venues (20%),^50^^,^^51^^,^^55^^,^^56^ two in local clinics (10%),^38^^,^^39^ and one at home (5%).^42^ One study was conducted both at home and in a local clinic (5%),^46^ while one study was conducted in three settings (a community venue, a clinic, and at school) (5%).^48^ One study was conducted in both the school and a community venue (5%).^49^ One study was conducted in a shopping mall (5%),^45^ and one did not report the setting (5%).^37^ See Table 2.

**Table 2:** Designs of included studies.

Primary author (Year); Country	Study design	Length of follow-up	Method of assessment	Population characteristics				Setting
				Child			Adults	
				Impairment/Disability; assessment method	Age range (years)	Sex	Parent/caregiver or community members	
Barros de Oliveira (2006); Brazil	One group before-after, no control	30 months	Observation	Multiple; Researcher determined	8 years and 3 months	All Female	1 caregiver	Community venues
Bharat (2021); India	One group before-after, no control	2 months	Quantitative surveys	Multiple; Not reported	0–6 years	Not reported	83 caregivers	Not reported
Chaudhry (2023); Pakistan	RCT with treatment as usual	No follow-up	Quantitative surveys and IDI	Multiple; Clinician determined	3–6 years	Not reported	Test = 13 caregivers Control = 13 caregivers Interviews = 7 parents	Clinic
Del Pilar Saa (2020); Guatemala	RCT, longitudinal mixed	2–3 months	Quantitative surveys and observation	Multiple; Not reported	18 months to 4 years	92% male	None	School and community venues
Estupiñan Vives (2017); Colombia	Programme evaluation	Not reported	Observation	Multiple; Not reported	7–9 years	Not reported	None	Community venues
Favazza (2016); Kenya	One group before-after, no control	No follow-up	Quantitative surveys and IDI	Intellectual, cerebral palsy and autism; Not specified	3.4–6.5 years	M = 14 F = 4	18 caregivers	School, clinic and community venues
Gebrael (2011); Brazil	Controlled before-after	Not reported	Quantitative surveys and observation	Visual; Researcher determined	4–6 years	Not reported	10 teachers	School
González Moreno (2017); Mexico	One group before-after, no control and programme evaluation	Not reported	Quantitative surveys and observation	Down syndrome; Self-report	7 years	Not reported	None	School
Gurgel (2014); Brazil	Qualitative	No follow-up	IDI	Not reported (recruited from a rehabilitation institute for children with disabilities)	3–6 years	M = 4	6	Clinic
Irawan (2023); Indonesia	One group before-after, no control	No follow-up	Quantitative surveys	Physical; Medical records and Pre-Screening Development Questionnaire	3–6 years	M = 8 F = 12	None	School
Jacob (2021); Nigeria	Controlled before-after	No follow-up	Quantitative surveys	Multiple; Clinician determined	Mean age = 11.6 years	Music (M = 8, F = 7) Pictorial (M = 8, F = 7) Control (M = 9, F = 8)	None	School
Lynch (2018); Malawi	Longitudinal mixed methods and programme evaluation	0.5 months	Mixed method	Visual; Researcher determined through registration records	0.75–6 years	M = 15 F = 15	26 caregivers	Home
Oliveira (2009); Brazil	Programme evaluation	Not reported	Quantitative surveys and IDI and FGD	Cerebral palsy; Not specified	5–10 years	All Male	None	Community venues
Pirnazar (2022); Iran	Controlled before-after	No follow-up	Quantitative surveys	Multiple; School file	8–12 years	All Female	None	School
Rerkmoung (2024); Thailand	RCT with treatment as usual	1 month	Quantitative surveys	Cerebral palsy; Not specified	7–2 years	Not reported	None	School
Rico-Olarte (2017); Colombia	One group before-after, no control	No follow-up	Quantitative surveys, IDI, observation	Multiple; Not specified	6–10 years	Not reported	None	Shopping mall
Ríos-Rincón (2016); Colombia	One group before-after, no control	1 month	Quantitative surveys	Multiple; Researcher determined	4–9 years	M = 3 F = 1	4 caregivers	Clinic and home
Rodríguez García (2023); Cuba	Programme evaluation	Not reported	Quantitative surveys and observation	Visual; Not specified	6–8 years	M = 5 F = 2	None	School
Sant (2024); India	One group before-after, no control	No follow-up	Quantitative surveys	Hemiplegic cerebral palsy; University of Michigan PSQ	4–12 years	F = 3 M = 3	None	School
Zerihun (2024); Ethiopia	Qualitative	No follow-up	IDI and FGD	Intellectual and autism; Not specified	2–9 years	M = 11 F = 8	19 caregivers, 15 community leaders	Community venues

IDI, in-depth interview; FGD, focus group discussion; RCD, randomized control trials.

### Sample and participant characteristics

A total of 215 children participated in the 20 studies, in addition to 181 caregivers and 39 community leaders, with an overall total of 432 participants. Over half of the studies had ten or fewer participants (*n* = 11; 55%),^39^^,^^44^, ^45^, ^46^, ^47^^,^^50^, ^51^, ^52^, ^53^^,^^55^^,^^56^ three studies had 11–20 participants (15%),^40^^,^^48^^,^^54^ four studies had 21–50 participants (20%),^38^^,^^41^^,^^43^^,^^49^ and two had 51 or more participants (10%).^37^^,^^42^

Most studies targeted children with multiple impairments (*n* = 13; 65%).^37^^,^^38^^,^^41^^,^^43^, ^44^, ^45^, ^46^, ^47^, ^48^^,^^50^^,^^51^^,^^55^^,^^56^ One study targeted children with physical disabilities (5%),^40^ two targeted children with intellectual disabilities (10%),^49^^,^^52^ and three targeted children with visual impairments (15%).^42^^,^^53^^,^^54^ One study did not specify the disability type.^39^

Of the included studies, 16 included children (80%),^40^^,^^41^^,^^43^, ^44^, ^45^^,^^47^^,^^50^, ^51^, ^52^, ^53^, ^54^^,^^56^ six studies (30%) included caregivers,^37^, ^38^, ^39^^,^^46^^,^^48^^,^^55^ and two studies included community leaders, such as teachers (15%).^42^^,^^49^

### Intervention characteristics

Just over half of the interventions were delivered by the researchers (*n* = 11, 55%),^39^, ^40^, ^41^, ^42^, ^43^, ^44^^,^^46^^,^^49^^,^^50^^,^^52^^,^^56^ with two studies including parents, and three studies involving parents and researchers in the intervention delivery (25%).^37^^,^^47^^,^^48^^,^^51^^,^^53^ One study (5%) involved community members,^55^ and two studies (10%) included specialised professionals, such as psychologists and speech therapists.^38^^,^^46^ Intervention characteristics are outlined in Table 3.

**Table 3:** Intervention components, delivery, and adaptation.

Primary author (Year); Country	Play components	Intervention delivery			Considerations^a^	
		Agent	Training provided	Duration (n = number of sessions)	Contextual	Cultural
Barros de Oliveira (2006); Brazil	Symbolic play, motor coordination games, and rule-based games	Parents/caregivers and community members	None	50 min per session (n = 84)	None	None
Baharat (2021); India	Mobile app with targeted play ideas.	Researcher and parents/caregivers	Yes—parents learned about child development	Not reported	None	None
Chaudhry (2023); Pakistan	Parents taught how to use play activities to enhance child's development e.g developmentally appropriate activities like passing a ball, and how to use what you have at home to play, such as a pan and spoons for noise, peek-a-boo games.	Psychologist	Not reported	60–90 min per session (n = 12)	Manual was translated into Urdu	Adapted illustrations used to retain cultural relevance
Del Pilar Saa (2020); Guatemala	Targeted skill development (motor skills, symbolic play, language development, social interaction, self-regulation, academic readiness) Activities undertaken in sensory-rich environments; Parental involvement: Monthly education sessions provided strategies for continuing intervention at home	Researcher	Not reported	3 h (n = 2–3)	Adapted to two different settings, process not reported	Not reported
Estupiñan Vives (2017); Colombia	Free-access role-play game kit “MoJi” designed for use in public spaces.	Parents/caregivers	Yes—instructions for assembly and use provided	Not reported	Designed to attract children and foster interaction in public spaces in Colombia	Not reported
Favazza (2016); Kenya	Sessions addressed fundamental motor skills, including opening and closing songs before and after a selection of 187 motor activities	Researcher and parents/caregivers	Yes–A train-the-trainer course included motor development, challenges in young children with intellectual disabilities, the curriculum, data collection, and programme adaptations. Trainers then trained site-level leaders using the same materials	30 min per session (n = 24)	None	Adapted to meet the needs of the child, family, and culture. Added the Kenyan national anthem as the opening song. Incorporated more kicking-related activities, as soccer is a popular sport
Gebrael (2011); Brazil	Play-based activities used to reinforce daily living skills, e.g. hygiene and nutrition Teachers trained to integrate toys and games into interactions with children to promote autonomy and independence	Not specified	Yes—No detail provided	90 min per session (n = 6)	None	Study reports cultural adaptation, process not reported
González Moreno (2017); Mexico	Group role-playing games for children with Down syndrome; activities included communication, social interaction, emotional expression, voluntary activity, object activity, and symbolic function	Researcher	Yes—Conducted by a specialist in child development	120 min per session (n = 130)	None	None
Gurgel (2014); Brazil	Play activities with caregivers and children with disabilities in a rehabilitation centre offered in 4 phases to build up to free play: awareness, action, contact, retraction.	Researcher	Not reported	Not reported	None	None
Irawan (2023); Indonesia	The use of Busy Books—interactive, sensorial books—for stimulation in fine motor development in preschool children	Researcher	Not reported	15 min per session (n = 4)	None	None
Jacob (2021); Nigeria	Music. Song with teacher, lyrics explained to children who were encouraged to sing along and dance. Child names inserted into the songs, actions included dancing, stamping PICTORAL–images and diagrams were drawn by hand using drawing books, pencils, and crayons. Children drew pictures representing their ideas and responses to questions	Researcher	Yes—3 days of training on music therapy delivered to teacher of children with special needs by therapist.	45 min per session (n = 24)	Songs were in the local language	None
Lynch (2018); Malawi	Information cards (n = 8) developed for caregivers with age appropriate play and communication activities for children between birth and 3 years with visual impairment. Caregivers used the cards at home. Low-cost locally sourced toys were also provided (e.g. a rattle to increase child's ability to stretch arm and reach).	Researcher	Yes—2-day training on the use of information cards and 1-day training on feasibility study	1 h per session (n = 12)	The Malawi Development Assessment Tool was used to align developmental activities with appropriate milestones	Line drawings of culturally appropriate mothers, family members, children with visual impairment and play objects were created.
Oliveira (2009); Brazil	Adaptation of toys, assistive devices for play, and assistive technology support	Researcher	Yes—No detail provided	Not reported	None	None
Pirnazar (2022); Iran	Playing games: clock and blow game to increase visual accuracy and concentration in a limited time Hopscotch to increase attention and concentration, gross motor coordination. Tic-tac-toe to increase concentration, attention, and accuracy in decision-making against the competitor. Figure and color games to improve visual and auditory concentration. Seeing and guessing games to promote visual discrimination and concentration.	Researcher	None	35 min per session (n = 16)	None	None
Rerkmoung (2024); Thailand	We Smile used skeletal tracking or motion capture to translate a child's movement on to the screen when they play a game. The player appears on the screen as cartoon character	Researcher	Yes—No detail provided	Duration of session not specified (n = 6)	None	None
Rico-Olarte (2017); Colombia	The HapHop-Physio mini-games included: selection games, writing games, reaction games, and repeating sequences games	Researcher	Yes—No detail provided	30 min per session (n = 1)	None	None
Ríos-Rincón (2016); Colombia	Children operated a Lego ‘robotic’ vehicle and played with their caregiver	Speech pathologist	Yes–Each mother was trained to operate, position, and reassemble the robot	15 min per session (n = 10)	None	None
Rodríguez García (2023); Cuba	Adapted traditional games, including activities that encourage mobility, spatial orientation, and interaction (e.g., games like “El tilín,” “Pelota rodada,” and “El laberinto”).	Researcher and parents/caregivers	None	Not reported	None	Traditional Cuban games were adapted for children with visual impairment
Sant (2024); India	Colouring pictures, drawing geometrical shapes, line joining drawings, thumbprints, and vegetable prints. Upper limb physical activities included clapping hands, moving the upper body, and rotating the neck and head	Parents/caregivers	None	60 min per sessions (n = 12)	None	None
Zerihun (2024); Ethiopia	Facilitated peer group programmeme for caregivers to learn about play. Based on principles of social communication interventions, developmental science, applied behaviour analysis, positive parenting and self-care methods and aligned with naturalistic developmental behavioural interventions	Researcher	Yes–Non-specialist facilitators trained over nine days on content of the sessions, home visits, goal-setting, and facilitation skills, with supervision and feedback from master trainers before independently leading the programmeme	Duration of sessions not specified (n = 9)	Involved facilitators who speak the local languages, understand the context and are part of the local community	

a Countries where no contextual or cultural considerations were undertaken apply to programmes from other settings transferred to those implementing the interventions.

All twenty interventions targeted children. Six interventions (30%) also targeted the caregivers of the children with disabilities,^37^^,^^38^^,^^41^^,^^42^^,^^48^^,^^49^ and two also targeted community leaders (10%).^41^^,^^54^

#### Contextual and cultural adaptation

Eight studies (40%) noted that specific adaptations were made to fit either the context or the culture of the targeted population.^38^^,^^41^^,^^42^^,^^48^^,^^50^^,^^51^^,^^53^^,^^54^ These adaptions were largely superficial adjustments rather than deeper, co-designed changes to the intervention's goals, delivery structure or underlying power dynamics. Contextual adaptations generally involved using local languages^38^^,^^41^ and fostering interactions in public spaces,^51^ as well as using local measures to align developmental activities with milestones considered appropriate in the population context.^42^ Cultural adaptations included adding the local national anthem as the opening song^48^ incorporating play activities popular in the region or using traditional games in the intervention,^53^ and adapting illustrations to retain cultural relevance.^42^ For example, in Pakistan, Chaudhry et al.^38^ adapted pictures of parents and children with Asian physical appearance, clothing, environment, and background pictures.

### Outcomes used in the studies

Nine studies (45%) employed multiple assessment methods.^38^^,^^45^^,^^48^^,^^50^^,^^52^, ^53^, ^54^^,^^56^ Seven studies (35%) used only quantitative questionnaires.^37^^,^^40^^,^^41^^,^^43^^,^^44^^,^^46^^,^^47^ Two assessment methods (10%) used only observations^51^^,^^55^ and two (10%) used only qualitative methods such as interviews or focus group discussions.^39^^,^^49^ Outcome reporting was predominantly around child development and functioning (n = 16, 80%),^37^^,^^38^^,^^40^^,^^41^^,^^43^, ^44^, ^45^, ^46^, ^47^, ^48^^,^^50^, ^51^, ^52^, ^53^, ^54^, ^55^ while half of the studies examined participation or social inclusion (n = 10, 50%).^38^^,^^39^^,^^42^^,^^43^^,^^46^^,^^49^^,^^51^^,^^53^^,^^55^^,^^56^ Fewer assessed caregiver or family well-being (n = 4, 20%).^37^, ^38^, ^39^^,^^54^ An outcome matrix is provided in Web Appendix A.

#### Intervention effectiveness, feasibility, and acceptability

Overall, studies reported positive outcomes (*n* = 15, 75%).^37^^,^^38^^,^^40^^,^^43^, ^44^, ^45^, ^46^^,^^48^^,^^50^, ^51^, ^52^, ^53^, ^54^, ^55^, ^56^ Two studies (10%) reported mixed outcomes.^39^^,^^47^ Studies that focused on testing the feasibility and acceptability of play-based interventions generally found the interventions to be both feasible and acceptable. However, some concerns were noted. For example, Sant et al.’s study^47^ in India and Gurgel et al.’s study^39^ in Brazil observed that not all parents felt comfortable with playing with their children, compared to children playing with each other.

Notably, only one of these studies conducted a cost analysis,^42^ which included the cost of the total intervention of the 6-month pilot (including all travel, allowances and toys) at approximately US$2420, with an average cost per child of US$82. The analysis did not include paid staff salaries, costs of consultants, costs of developing training materials, or family costs associated with these activities. There were additional costs that should also be considered. Table 4 provides details on the outcomes, feasibility, acceptability, and effectiveness of the interventions.

**Table 4:** Intervention outcomes and implications.

Primary author (Year); Country	Data collection measures			Outcomes	Implications
	Intended outcomes	Measures	Time assessed		
Barros de Oliveira (2006); Brazil	Quality of life Interpersonal interaction	Improvements in motor coordination, social interaction, communication, and autonomy	Pre- and post-intervention	Clinical effectiveness: Improvements in constructive and fine motor praxes; Enhanced social interaction, autonomy, and self-image. Feasibility: Sessions were effectively carried out over the study period. Acceptability: Both child and caregiver actively participated.	Play activities promote motor coordination, autonomy, and social interaction, with potential applications in therapeutic and educational contexts.
Bharat (2021); India	Psychological/Emotional Interpersonal interaction	Parental Stress Index	Pre-, mid-, and post-intervention	Clinical effectiveness: Lower parental stress (PSI scores) Children's developmental progress improved in parents with lower PSI scores.	Addressing parental anxiety may improve interaction with the child.
Chaudhry (2023); Pakistan	Physical Psychological/Emotional Quality of life Cognitive development Interpersonal interaction	PHQ-9 GAD-7 Euro-Qol-5 Dimensions Parenting Stress Index Vineland Adaptive Behaviour Scales Individual interviews	Pre- and post-intervention	Clinical effectiveness: Statistically significant lower PHQ-9, GAD-7, and PSI. Statistically significant increase in quality of life. Statistically significant increase in socialisation, increase in communication, daily living, socialisation, and motor skills.	The culturally adapted parenting intervention was well-tolerated by mothers with probable depression of young children with ID.
Del Pilar Saa (2020); Guatemala	Psychological/Emotional Cognitive development	Bayley-III cognitive, fine motor, and gross motor subtests	Pre- and post-intervention	Feasibility: PITBJ is feasible to implement in Guatemala. Attendance frequency and variability in caregiver training and support were limitations Acceptability: Caregivers were actively involved through education sessions.	PITBJ programmes have a positive impact on children's functional development, enhancing adaptive, motor, cognitive, and socio-emotional skills.
Estupiñan Vives (2017); Colombia	Psychological/Emotional Interpersonal interaction	Improvement in social interaction, imagination, autonomy, and fine motor skills.	Post-intervention	Clinical effectiveness: Enhanced social inclusion, psychological development, and motor skills. Feasibility: The kit is accessible and easy to use. Acceptability: Participants demonstrated engagement and positive feedback.	The kit supports inclusion, autonomy, and creativity for children with IDD.
Favazza (2016); Kenya	Physical	Test of Gross Motor Development	Pre- and post-intervention	Clinical effectiveness: all children improved on their motor abilities. Statistically significant difference from preintervention to postintervention on the Gross Motor Quotient (t = 12.44, p < 0.001). Statistically significant difference between the preintervention and postintervention on Locomotion (t = 9.23, p < 0.001) and Object Manipulation (t = 12.96, p < 0.001) subtests Feasibility: All children were present for 90% or more of the YA lessons. Acceptability: Yes, but no details reported.	There is need to measure impact of motor programmes like YA on other areas of development, and the global context for using motor play programmes as a gateway to receiving developmental screening and access to preschool.
Gebrael (2011); Brazil	Quality of life Interpersonal interaction	Increased teacher strategies and improved child autonomy in hygiene and nutrition tasks.	Pre- and post-intervention	Clinical effectiveness: Improved teacher strategies and increased student autonomy in hygiene and nutrition tasks. Feasibility: Teachers completed the programme and reported positive feedback. Acceptability: Teachers found the programme relevant and beneficial.	Collaborative consultation in occupational therapy can effectively prepare teachers for inclusive education, fostering independence in children with low vision.
González Moreno (2017); Mexico	Physical Interpersonal interaction	Psychological development indicators (communication, social skills, symbolic functions).	Pre- and post-intervention	Clinical effectiveness: Gains in communication, symbolic thinking, and voluntary actions in the experimental group compared to control. Feasibility: Demonstrated through successful implementation. Acceptability: Positive feedback from participants.	Role-play group interventions can enhance psychological and social development in children with Down syndrome, promoting inclusion in educational and social settings.
Gurgel (2014); Brazil	Interpersonal interaction	Not reported	Post intervention	Clinical effectiveness: Phase 1– Despite play having been proposed to the adults, during this phase all took advantage of the fact that they had a psychologist researcher, to clear out doubts and share problems and difficulties, besides showing disinterest and lack of experience in playing. Phase 4—No concerns raised by caregivers about their children. Children acted independently and interacted more freely with everyone.	New ways of relating emerged, which extended beyond predefined diagnostic categories
Irawan (2023); Indonesia	Physical	Pre-Screening Questionnaire (Indicating fine motor development)	Pre- and post-intervention	Clinical effectiveness: Statistically significant improvement in fine motor skills. The results of the pre-test showed that out of 20 participants, 11 participants had deviant fine motor development (55%). The post-test showed that out of 20 participants, 17 participants (85%) had proper fine motor development. In the intervention group, the average fine motor development of children before busy book stimulation was 62.47 ± 7.539, while after busy book stimulation it increased to 86.08 ± 4.104. The statistical test results of the Wilcoxon signed ranks test obtained a p-value 0.000 (p-value <0.05).	Not specified
Jacob (2021); Nigeria	Cognitive development	Moss Attention Rating Scale	Pre- and post-intervention	Clinical effectiveness: Significant statistical difference between pre-test and post-test results. There was statistically significant main effect of treatment in enhancing attention span of children with intellectual disability [*F*_(2_, _35)_ = 443.582; p < 0.05; partial η^2^ = 0.962]. The effect size is 96.2%. This implies that 96.2% variance in the post–reading performance of learners with intellectual disability was accounted for by treatment, hence, there was a considerable difference in the attention span of children with intellectual disability. Pictorial illustration had the highest post-attention score, followed by music therapy, while post attention span scores for the control group were the least.	The use of the two strategies (music therapy and pictorial illustration) to enhance the attention of children with intellectual disability should be encouraged among their teachers.
Lynch (2018); Malawi	Interpersonal interaction	Reported in individual interviews and an FGD	Post intervention	Clinical effectiveness: Improved confidence and enabling the child to explore. Improved understanding and communication with the child. Being able to integrate play into everyday activities (with some difficulty). Feeling enabled to be more accepting of their child. Improved relationships with the community. Limited improvement in trusting other carers/nurseries. Cost analysis: Total intervention cost for 6-months (for travel, allowances and toys) was US$82 per child. Analysis did not include paid staff salaries, costs of consultants, costs of development of training materials or family costs associated with these activities.	Integrating provisions of advice and information to carers of children with VI in the early years into the Care for Child Development (WHO) but could be expanded to include children with other disabilities.
Oliveira (2009); Brazil	Physical Psychological/Emotional	Enhanced play quality, reduced dependence, increased interaction and satisfaction.	Pre- and post-intervention	Not reported	Assistive technology significantly improves the quality of play and life for children with cerebral palsy.
Pirnazar (2022); Iran	Cognitive development	Toulouse–Pieron test Cognitive Diagnostic Battery	Pre- and post-intervention	Clinical effectiveness: Mean attention span differed after the intervention sessions (p = 0.001). The Mean ± SD and standard deviation of attention span obtained from the TPT before the therapeutic sessions in two groups were 7.60 ± 4.72 and 6.1 ± 6.85, respectively, which changed to 18.78 ± 4.84 and 6.50 ± 3.86 after the intervention, respectively. 94% of the variation in the attention span of the experimental group was due to participation in the therapeutic sessions. 29% of the variation in the attention span of the experimental group was due to attending play therapeutic sessions.	Children who are given more opportunities to repeat their games may show greater levels of attention and participation.
Rerkmoung (2024); Thailand	Psychological/Emotional	Gait speed (m/s), Cadence (step/min), Stride length (m), Step length (m), Paediatric Balance scale, GMCFS, Balance path length with eyes opened and closed (mm), Balance surface area with eyes opened (mm^2^) and Balance surface area with eyes closed (mm^2^).	Pre- and long-term follow-up	Clinical effectiveness: After 8 weeks of training, gait speed in intervention group increased significantly 0.071 (p = 0.043). In the between-group comparison, the balance surface area with eyes opened at 8th week in the intervention group was significantly better (p = 0.028). No adverse event was found in both groups. Feasibility: Yes, but no details reported.	Not reported
Rico-Olarte (2017); Colombia	Cognitive development	Observation Smiley-o-meter Again–Again Table System Usability Scale	Pre- and post-intervention	Acceptability: System Usability Scale = 87.5 out of 100.	Not reported
Ríos-Rincón (2016); Colombia	Interpersonal interaction	Test of Playfulness Canadian Occupational Performance Measure	Pre-intervention, Post-intervention, Mid-intervention and Long-term follow-up	Clinical effectiveness: The findings support to the play theories and approaches that explain play from a psychobiological perspective (optimal arousal) and from a cognitive and social perspective. Statistical comparisons using the 2 SD band and X-moving range chart methods revealed that all the children's levels of playfulness increased considerably while they played with the robot. Comparison of baseline and follow-up phase indicated that three children had retention of improved level of playfulness. Acceptability: Acceptable, but not reported.	Not reported
Rodríguez García (2023); Cuba	Physical Interpersonal interaction	Not specified	Pre- and post-intervention	Clinical effectiveness: Enhanced mobility, spatial orientation, and confidence among visually impaired children, which facilitated inclusion in school and extracurricular activities. Feasibility: Indicated by successful implementation. Acceptability: Families and children reported positive engagement and benefits from the adapted games.	Culturally relevant and adapted traditional games may improve the mobility, spatial orientation, and inclusion of visually impaired children in social and educational contexts, especially post-pandemic.
Sant (2024); India	Quality of life	Sleep quality	Pre- and post-intervention	Clinical effectiveness: Increased sleep quality after 4 weeks. The median pretest and post-test PSQ scores were 0.68 and 0.49, respectively. Interquartile Range (IQR) value for the outcome measure was 0.0675 and 0.0775. Feasibility: Five participants discontinued the protocol at the end of the 3rd and 4th weeks. Acceptability: Participants failed to conduct the protocol due to a lack of cooperation from the parents.	Art therapy can be integrated into physiotherapy interventions and incorporated in daily activities to improve sleep quality in children with hemiplegic cerebral palsy.
Zerihun (2024); Ethiopia	Quality of life Interpersonal interaction	Not reported	Post-intervention	Feasibility: Feasible, but not all have access to toys and barriers reported were attending the group session on time (transportation, family responsibilities). Acceptability: Yes, however not all felt comfortable with playing with their children	The CST programme has the potential to be scaled up for families of children with developmental disabilities in Ethiopia and other low-resource contexts.

### Grey literature

From the 50 sources reviewed, ten were considered eligible for inclusion.^57^, ^58^, ^59^, ^60^, ^61^, ^62^, ^63^, ^64^, ^65^, ^66^ These materials included factsheets, guides, books, and a video. Most resources had a shared goal of demonstrating how play can help the development and inclusion of children with disabilities. All eligible sources discussed the importance of play in helping children develop, both physically and socially. Eight sources (80%) gave specific strategies for making play more accessible for children with disabilities. For example, UNICEF, Sense,^59^ Disability Africa,^64^ Raisingchildren. net.au,^60^ and Cognitive Behavioural Play Therapy^65^ all highlighted how play can help reduce isolation and build skills such as communication and coordination.

Most resources (*n* = 7; 70%) focused on providing tools to parents, caregivers, or teachers to make play accessible for all children. Practical resources such as the Ndinogona “I Can” programme by Shonaquip^66^ and the Physiopedia informational guides^62^^,^^63^ included hands-on guides that give ideas and advice for working with children with disabilities who might need extra support to engage in play. Six resources (60%) emphasised that play is not just key for learning but also for inclusion, and make the case that every child has a right to play. Some (*n* = 4; 40%) also emphasised that giving caregivers and professionals the tools to make play accessible is just as important. Overall, all resources shared the rationale that when children with disabilities have the opportunity to play, they grow in confidence, break out of isolation, and gain the same opportunities as any other child.

While all resources targeted caregivers and educators, several aimed for a wider audience. For example, the book on inclusive play, ‘*Guidelines for supporting children with disabilities' play’*, also spoke to policymakers and toy manufacturers.^61^ Meanwhile, Disability Africa's guide^64^ had a strong focus on helping NGOs and community leaders in poorer settings with ideas and plans on how to best include children with disabilities. The content and format of the resources also varied. Some, like the UNICEF factsheet, were short and simple, while others, Shonaquip's guide, went deeper, offering training and support ideas.^57^^,^^66^ Additionally, the materials targeted different disabilities. For example, Physiopedia's guides were targeted to support children with cerebral palsy or severe disabilities, giving specific advice.^62^^,^^63^ On the other hand, the factsheet from Sense included broader information and did not go into as much detail for particular disabilities.^59^ Cognitive Behavioural Play Therapy^65^ was another resource that provided insights into how play-based techniques can be used to support children with emotional and behavioural difficulties, demonstrating its adaptability across various therapeutic contexts. None of the resources identified mention cultural adaptations.

## Discussion

This review included 20 studies that explored play-based interventions for children with disabilities across different LMIC settings and described different types of play interventions and support initiatives, including practical guides available online on play, interventions on targeted play for children with disabilities, caregivers and community members.

The research studies mainly focused on play as a way to facilitate therapy, communication, and skill development, especially for children with cerebral palsy and neurodevelopmental delays. Parental anxiety and stress, and how best to support caregivers of children with multiple impairments, were also addressed in play programmes in Pakistan^38^ and India.^37^ They all also looked at the impact of the intervention on children. Not all articles reported outcomes or used quantitative assessment, but those that did reported on the benefits of play for children with disabilities, including the study by Barros de Oliveira et al. (2006)^55^ that reports on play activities promoting motor coordination, autonomy, and social interaction, and González Moreno et al.’s study (2017)^52^ that reports on how role-play group interventions can enhance psychological and social development in children with Down Syndrome. Evidence suggests that effective care for children with disabilities requires awareness, better detection, early support, social-emotional learning, and improved access to treatment.^67^, ^68^, ^69^ This review highlights potential play interventions that can integrate these factors, though the evidence remains limited.

The study interventions were set in different locations, such as schools, community venues, clinics, and homes, demonstrating a wide range of possible settings in which to engage children with disabilities in play. Most studies were conducted in schools,^37^^,^^40^^,^^41^^,^^43^^,^^47^^,^^52^, ^53^, ^54^ while only one was conducted at home.^42^ Challenges to implementing play programmes related to parental engagement and cultural attitudes towards play, such as parents’ discomfort with playing with their children.^39^^,^^47^

A key gap identified in our review was the lack of economic evaluation, limiting the possibility of drawing conclusions regarding the financial viability of play-based interventions for children with disabilities. This finding aligns with a recent scoping review which mapped economic evaluations of caregiver interventions for children with developmental disabilities and similarly highlighted the limited evidence and methodological challenges in this area.^70^ While emerging data from some common developmental disabilities suggest potential for cost-saving and cost-effective caregiver interventions that improve quality of life for both children and their families, these insights have not yet been extended to play-based approaches. In low-resource settings, governments and policymakers may face difficult decisions between funding targeted disability initiatives and strengthening broader early childhood development systems. Integrating play-based interventions into existing platforms could present a cost-saving opportunity, but the extent of this benefit remains unknown.

The findings from this review fit with wider research on how play supports child development and inclusion for children with disabilities. Play is widely recognised as important for cognitive, social, and motor development^34^^,^^71^ and structured and adapted play interventions can help improve social interaction, communication skills, and motor abilities in children with disabilities.^72^ Community involvement in supporting play may contribute to the effectiveness of interventions.^73^

Nevertheless, while play is a feature of childhood and many developmental principles transcend cultural borders, the very need for play programmes, and the degree to which they should be adapted, must be established through dialogue with the community. Perspectives on play and its value are shaped by broader social and economic realities^17^ and when play for children with disabilities is warranted, evidence shows it works best when it resonates with families' existing practices.^74^^,^^75^ Best-practice guidance—exemplified by Bernal's Ecological Validity Framework^76^^,^^77^ and the FRAME (Framework for Reporting Adaptations and Modifications to Evidence-based Interventions) checklist^78^ —emphasises participatory co-design with local caregivers, iterative piloting, and transparent reporting of who modified each component, why and when. In the studies we reviewed, however, “adaptation” was typically limited to surface changes such as re-creating images, translating or simplifying text, and adding familiar games or songs, with little explanation of the decision-making behind these changes. Interventions that are co-developed with local communities to reflect their values and priorities mitigate the ethical concerns of introducing caregiving practices that do not align with those of the communities, such as overriding them with Western-based models.^15^ Interventions should not focus on changing caregiver beliefs around play, but rather on creating more accessible opportunities for children with disabilities to play within their cultural structures.^15^^,^^79^ Ignoring local practices or treating them as “deficient” simply because they differ from Western norms also perpetuates epistemic injustice.^80^ Recognising these dynamics, and documenting adaptions in depth, makes programmes that integrate local traditions and community-driven approaches more culturally respectful and ultimately more sustainable and impactful.^15^^,^^80^

As 80% of people with disabilities live in LMICs,^81^ there is an urgent need for play solutions that fit these contexts. Using data from high-income countries can be ineffective, especially when approaches do not align with local cultures or resources.^79^ This review highlights how they could be adapted for children with disabilities, such as through community-led initiatives or integrating play into daily caregiving routines.^15^^,^^17^ More evidence is needed on how to adapt and implement these interventions effectively in different countries.

Importantly, we emphasise that play must be recognised first and foremost as a fundamental right, as enshrined in the UN Convention on the Rights of the Child (UNCRC), not merely as a developmental intervention. A rights-based approach demands that all children, including those with disabilities, are entitled to play for its own sake, because it is fun and foundational to being a child. Framing play only in terms of therapeutic or functional outcomes risks reinforcing deficit-based views of disability, where value is tied to productivity or measurable change. Instead, valuing play as an expression of agency and identity challenges assumptions that the experiences of children with disabilities must be justified through outcomes.

Our scoping review has several strengths. We undertook a systematic and rigorous approach with predefined inclusion criteria, two independent reviewers, and structured data extraction to ensure reproducibility and minimise bias. Consequently, our review provides a comprehensive overview of the existing literature on play for children with disabilities from diverse sources. Our review also incorporates research that has been published in several languages, enabling integration of research from Latin America where a large number of studies have been published in Portuguese or Spanish. This review also has limitations to consider when interpreting the results. We were unable to access four full-text articles for inclusion, despite our attempts to contact the corresponding authors. The age range of children (2–12 years) of the eligible studies encompasses different developmental stages. While this is not necessarily a drawback, readers should be aware that some play programmes targeted children who were considerably younger than twelve years, while others target those who are closer to twelve years. Interventions that have the possibility of being successful for young children may not be as successful for older children due to their distinct developmental requirements and potential differences in response. We were unable to discern the type of school at which the school-based interventions took place, and this limits our ability to analyse the possible difference between school settings. Additionally, the studies did not provide information on the impact of some of the cultural and contextual adaptations made.

Our findings have important implications for future research. Disability research has grown since the 2006 UN Convention on the Rights of Persons with Disabilities,^2^ with ongoing calls for scalable disability inclusive interventions for children. However, despite the increased focus on child health and disability rights, this review suggested that play support for children with disabilities remains overlooked in research. There are a lack of studies on the long-term effects of play-based interventions. We only identified studies that assessed outcomes over a short duration. Many of the studies had a small sample size, with half of the studies including ten participants or fewer. Future research should focus on larger samples. Additionally, only one study attempted a cost analysis, and even then, it did not cover all costs (aspects such as the long-term costs of play resources or the financial impact on families were left out). Studies that include a cost analysis are needed. Another gap identified was the lack of research on play interventions for children with hearing impairments. The review found that most of the interventions were led by researchers, whilst some engaged parents, community members, or specialists such as speech therapists and psychologists to facilitate the activities. There is also a need for more community-driven research. Getting local communities more involved, whether in designing interventions or running them, could make them far more relevant and acceptable. Future research should focus on the practical application of lessons learned and how programmes can be run in the community. Only nine studies adapted their interventions to reflect cultural and contextual factors, incorporating local languages, traditional games, and familiar imagery. However, these studies gave minimal details on how they considered cultural appropriateness. Some mentioned the language was adapted, without details on other cultural adaptations. Thus, future research should be more specific on good practices for adaptation. Future studies should also employ more rigorous and transparent methodologies, including the use of control groups, validated outcome measures, and mixed methods designs to capture both quantitative and experiential outcomes. Researchers should also document more clearly the processes of cultural adaptation, including who was involved, how decisions were made, and which aspects of the intervention were modified, to improve replicability and contextual relevance. Lastly, there is an importance to abide by local developmental milestones and means of assessing disability severity, if available.

Play-based programmes must be grounded in the cultural, social, and relational contexts of the targeted populations. While a few studies included surface-level adaptations, such as translated materials or the use of traditional songs, these do not constitute meaningful cultural integration. True relevance requires that programmes be co-designed with those who hold deep, lived knowledge of the communities involved, including caregivers, local practitioners, and the children. Rather than imposing externally defined models of development or disability, programmes should respect and reflect local understandings of childhood, play, and inclusion. This includes recognising the essential roles that extended family and community members play in children's lives and acknowledging that the right to play, as outlined in the UNCRC, belongs to all children and must be supported by the social networks that surround them. Future research should identify effective strategies for adapting play interventions, while also respecting local understandings of play. Additionally, practice should prioritise collaborative, context-led development of interventions that promote not just effectiveness, but dignity, agency, and belonging for children with disabilities.

This review explored play-based interventions for children with disabilities in LMICs. The studies included highlight the potential of these interventions to enhance therapy, communication, and social inclusion. Gaps remain in understanding their feasibility and scalability. Cultural and contextual adaptations were inconsistently reported and the lack of economic evaluations limit the understanding of how play interventions for children with disabilities can be implemented in low-resource settings. Future studies should explore how prioritising inclusive, culturally relevant, and economically feasible approaches in play-based interventions can be leveraged to improve the well-being and development of children with disabilities.

## Contributors

All the authors contributed to the conceptualising of the scoping review and contributed significantly to writing the manuscript. VR and AvdW conducted systematic searches of the databases, completed the full-text screening and data extraction. VR wrote the full first draft manuscript with assistance from AvdW. TS was the second reviewer for the titles and abstract screening and served as the third reviewer in resolving conflicts following full-text review and data extraction. VR, AvdW and TS accessed and verified the underlying data.

## Data sharing statement

The data used in this scoping review are existing literature and publicly available.

## Declaration of interests

The authors declare that they have no conflict of interests.

## Appendix A Supplementary data

Supplementary Materials

LNCaP STR
