# Supplementary material for: Mapping play-based interventions for children with disabilities in LMICs: a scoping review on cultural relevance, implementation, and impact
Source: eClinicalMedicine. 2025 Sep 2;88:103444. doi: 10.1016/j.eclinm.2025.103444 (PMC12441714; doi:10.1016/j.eclinm.2025.103444)
Supplement: Supplementary Materials [file mmc1.docx]

Web appendix A

| **Outcome domain** | **n / 20 studies* (% of total)** | **Illustrative examples** |
| --- | --- | --- |
| **Child development & functioning** (motor, cognitive, emotional, quality of life) | 16 (80%) | • Gross- and fine-motor gains following a fundamental-motor play curriculum in Kenya (Favazza 2016)  • Improvements across motor, communication, daily-living and cognitive skills in a parent-mediated programme in Pakistan (Chaudhry 2023) |
| **Participation / social inclusion** (interaction, autonomy, inclusion) | 10 (50%) | • Role-play “MoJi” kit enhanced social inclusion and autonomy in Colombia (Estupiñan Vives 2017)  • Caregiver-led play cards strengthened community interaction and acceptance in Malawi (Lynch 2018) |
| **Caregiver & family well-being** (stress, depression, confidence) | 4 (20%) | • Mobile-app–guided play ideas lowered Parenting Stress Index scores in India (Bharat 2021)  • Parenting-with-play sessions reduced maternal depression, anxiety and stress in Pakistan (Chaudhry 2023) |
| **Implementation outcomes** (feasibility, acceptability, cost, usability) | 13 (65%) | • ≥ 90 % session attendance demonstrated high feasibility for the Kenyan motor-skills programme (Favazza 2016)  • Six-month early-vision-impairment play intervention costed at ~US $82 per child in Malawi (Lynch 2018) |
