# Supplementary material for: Mapping play-based interventions for children with disabilities in LMICs: a scoping review on cultural relevance, implementation, and impact
Source: eClinicalMedicine. 2025 Sep 2;88:103444. doi: 10.1016/j.eclinm.2025.103444 (PMC12441714; doi:10.1016/j.eclinm.2025.103444)
Supplement: LNCaP STR [file mmc2.docx]

**Medline, Embase, PsycINFO search terms***

**Disability**

((disable* or Disabilit* or Handicap*) adj2 (child* or infant* or young person)).tw.
(Physical* adj2 (impair* or deficienc* or disable* or disabili* or handicap*)).tw.
((Cerebral pals* or Spina bifida or Muscular dystroph* or Arthriti* or Osteogenesis imperfecta or Muscul* abnormalit* or Skeletal abnormalit* or Limb abnormalit* or Amputation* or Clubf$$t or Poliomyeliti* or Paraplegi* or Paralys* or Paralyz* or Hemiplegi*) adj2 (child* or infant* or young person)).tw.
((Hearing or Acoustic or Ear$3) adj2 (loss* or impair* or deficienc* or disable* or disabili* or handicap*)).tw.
((Visual* or Vision or Eye$3) adj2 (loss* or impair* or deficienc* or disable* or disabili* or handicap*)).tw.
((Deaf* or Blind*) adj2 (child* or infant* or young person)).tw.
exp Hearing impairment/ or exp vision disorders/ or exp Blindness/
((Intellectual* or Mental* or Psychological* or Developmental) adj2 (impair* or retard* or deficienc* or disable* or disabili* or handicap* or ill?6)).tw.
(epilepsy or seizure*).tw.
((Cogniti* or mental*) adj2 (impair* or deficienc* or disable* or disabili* or handicap*)).tw.
((communication or language or speech or learning) adj2 disorder*).tw.
(Autis* or Dyslexi* or Down* Syndrome or Mongolism or Trisomy 21).tw.

**Low-and-middle income countries**
developing country/ or middle income country/ or LMICs.mp. or low income country/
.nlpx
(Afghanistan or Albania or Algeria or Angola or Argentina or Armenia or Azerbaijan or Bangladesh or Belarus or Belize or Benin or Bhutan or Bolivia or "Bosnia and Herzegovina" or Botswana or Brazil or Bulgaria or "Burkina Faso" or Burma or Burundi or "Cabo Verde" or Cambodia or Cameroon or "Central African Republic" or Chad or China or Colombia or Comoros or "Congo, Dem. Rep." or "Congo, Rep." or "Costa Rica" or "Côte d'Ivoire" or Cuba or Djibouti or Dominica or "Dominican Republic" or Ecuador or Egypt or "El Salvador" or "Equatorial Guinea" or Eritrea or Eswatini or Ethiopia or Fiji or Gabon or Gambia or Georgia or Ghana or Grenada or Guatemala or Guinea or "Guinea-Bissau" or Guyana or Haiti or Honduras or India or Indonesia or Iran or Iraq or Jamaica or Jordan or Kazakhstan or Kenya or Kiribati or "Korea, Dem. People's Rep." or Kosovo or "Kyrgyz Republic" or Lao or Lebanon or Lesotho or Liberia or Libya or Madagascar or Malawi or Malaysia or Maldives or Mali or "Marshall Islands" or Mauritania or Mauritius or Mexico or Micronesia or Moldova or Mongolia or Montenegro or Morocco or Mozambique or Myanmar or Namibia or Nauru or Nepal or Nicaragua or Niger or Nigeria or "North Macedonia" or Pakistan or Palau or Palestine or Panama or "Papua New Guinea" or Paraguay or Peru or Philippines or Rwanda or Samoa or "São Tomé and Principe" or Senegal or Serbia or Seychelles or "Sierra Leone" or "Solomon Islands" or Somalia or "South Africa" or "South Sudan" or "Sri Lanka" or "St. Kitts and Nevis" or "St. Lucia" or "St. Vincent and the Grenadines" or Sudan or Suriname or "Syrian Arab Republic" or Tajikistan or Tanzania or Thailand or "Timor-Leste" or Togo or Tonga or Tunisia or Turkey or Turkmenistan or Tuvalu or Uganda or Ukraine or Uzbekistan or Vanuatu or Venezuela or Vietnam or "West Bank and Gaza" or Yemen or Zambia or Zimbabwe).mp. [mp=title, abstract, heading word, drug trade name, original title, device manufacturer, drug manufacturer, device trade name, keyword heading word, floating subheading word, candidate term word]

**Play**
play/ or play therapy/

**Publication date**

2020 to current

*These were translated and adapted to Spanish and Portuguese for the LILACS search
